# Investigation of tumor and vessel motion correlation in the liver

**DOI:** 10.1002/acm2.12943

**Published:** 2020-06-13

**Authors:** Sydney A. Jupitz, Andrew J. Shepard, Patrick M. Hill, Bryan P. Bednarz

**Affiliations:** ^1^ Department of Medical Physics University of Wisconsin‐Madison Madison WI USA; ^2^ Department of Human Oncology University of Wisconsin‐Madison Madison WI USA

**Keywords:** breathing motion, IGRT, liver, motion management, vessel tracking

## Abstract

Intrafraction imaging‐based motion management systems for external beam radiotherapy can rely on internal surrogate structures when the target is not easily visualized. This work evaluated the validity of using liver vessels as internal surrogates for the estimation of liver tumor motion. Vessel and tumor motion were assessed using ten two‐dimensional sagittal MR cine datasets collected on the ViewRay MRIdian. For each case, a liver tumor and at least one vessel were tracked for 175 s. A tracking approach utilizing block matching and multiple simultaneous templates was applied. Accuracy of the tracked motion was calculated from the error between the tracked centroid position and manually defined ground truth annotations. The patient’s abdomen surface and diaphragm were manually annotated in all frames. The Pearson correlation coefficient (CC) was used to compare the motion of the features and tumor in the anterior–posterior (AP) and superior–inferior (SI) directions. The distance between the centroids of the features and the tumors was calculated to assess if feature proximity affects relative correlation, and the tumor range of motion was determined. Intra‐ and interfraction motion amplitude variabilities were evaluated to further assess the relationship between tumor and feature motion. The mean CC between the motion of the vessel and the tumor were 0.85 ± 0.11 (AP) and 0.92 ± 0.04 (SI), 0.83 ± 0.11 (AP) and −0.89 ± 0.06 (SI) for the surface and tumor, and 0.80 ± 0.17 (AP) and 0.94 ± 0.03 (SI) for the diaphragm and tumor. For intrafraction analysis, the average amplitude variability was 2.47 ± 0.77 mm (AP) and 3.14 ± 1.49 mm (SI) for the vessels, 2.70 ± 1.08 mm (AP) and 3.43 ± 1.73 mm (SI) for the surface, and 2.76 ± 1.41 mm (AP) and 2.91 ± 1.38 mm (SI) for the diaphragm. No relationship between distance and motion correlation was observed. The motion of liver tumors and liver vessels was well correlated, making vessels a suitable surrogate for tumor motion in the liver.

## 1 INTRODUCTION

Intrafraction motion due to respiration can impact the accuracy of external beam radiation therapy. Managing intrafraction motion remains problematic especially in the thoracic and abdominal regions. Tumor motion during treatment has been reported up to 20 mm in the lung, pancreas, and kidneys, and up to 30 mm in the liver.^1^, ^2^ Tumor motion cannot be predicted by tumor size or location, so direct or indirect tumor tracking is often utilized in the clinic when appropriate.^3^ Tumor‐tracking strategies are implemented to reduce margins placed around the tumor and further reduce absorbed dose to healthy tissue.

Most often, clinics employ systems that rely on external or internal surrogate fiducials as correlates of tumor motion. External motion tracking systems monitor the motion of the chest or abdomen through optical tracking whereas internal surrogate fiducials, such as implanted gold fiducial seeds, clips, or electromagnetic beacon transponders, are surgically implanted prior to treatment and subsequently monitored using x‐ray fluoroscopy or electromagnetic arrays. The accuracy of systems that use external or internal fiducials will depend on how well the motion of the fiducial(s) correlates with the motion of the tumor. In the case of external fiducials, reported correlations between internal fiducial implants and external markers have varied. Although good correlations were observed in thoracic^4^ and abdominal regions,^5^, ^6^ others have concluded that external surrogates are not sensitive enough to capture breathing variability.^7^, ^8^, ^9^, ^10^, ^11^ For these types of surrogates, issues with phase mismatch, internal drift, and deformation have been reported.^7^, ^8^, ^9^, ^10^ Correlations tend to improve for fiducials implanted close to tumors.^11^ However, implanting fiducials is invasive and introduces additional risks to the patient. Once implanted, fiducials only provide a point‐wise estimate of the tumor volume location and may be subject to significant drift over the course of the treatment.

Internal tracking is possible with in‐room or on‐board imaging systems. The Cyberknife® Tracking System uses ceiling mounted kV x‐ray sources to produce real‐time digitally reconstructed radiographs (DRR). However, this approach adds absorbed dose to the patient. Magnetic resonance (MR)‐guided delivery systems such as MRIdian (ViewRay, Inc., Cleveland, OH, USA) and Unity (Elekta, Stockholm, Sweden) can image thoracic and abdominal tumor motion without exposing the patient to additional absorbed dose. However, even when these costly systems are available, there are situations when the tumor cannot be imaged directly due to poor MR contrast. In these scenarios, it may be desirable to use other anatomical features that provide better MR contrast.^12^ While Yang et al. concluded that liver tumor motion and diaphragm motion were well correlated when their separation distance was small,^13^ others have shown that liver tumor motion does not correlate well with diaphragm or abdominal wall motion.^14^, ^15^ Liver vessels offer another alternative for feature tracking as the liver is highly vascular and blood vessels provide suitable MR contrast, especially the large hepatic portal vein.^12^

Multiple studies have investigated correlations between the tumor and internal anatomical surrogates in the thoracic and abdominal region.^13^, ^15^, ^16^, ^17^, ^18^, ^19^, ^20^, ^21^ Schlosser et al.^21^ showed results from healthy volunteers indicating that monitoring motion based on internal features close to a target may be more accurate than an external marker, yet the study fell short of using tumors from patient data. To the best of our knowledge, no studies have been published investigating liver vessels as possible surrogates for tumor motion with patient data. Despite the dearth of literature on vessel–tumor correlation, multiple groups have worked on developing and implementing vessel tracking techniques or vessel‐based tumor tracking techniques.^22^, ^23^, ^24^, ^25^, ^26^, ^27^, ^28^, ^29^, ^30^ This work aims to fill the gap in the literature connecting vessel and tumor motion in the liver for the purpose of imaging‐based motion management. By comparing the motion correlation between the tumor and vessels to that of the external surface and of the diaphragm, vessel tracking can be evaluated in comparison to the current practice of external surface tracking and to another potentially viable surrogate.

## 2 MATERIALS AND METHODS

### 2.A 2D image data

**Fig. 1:**
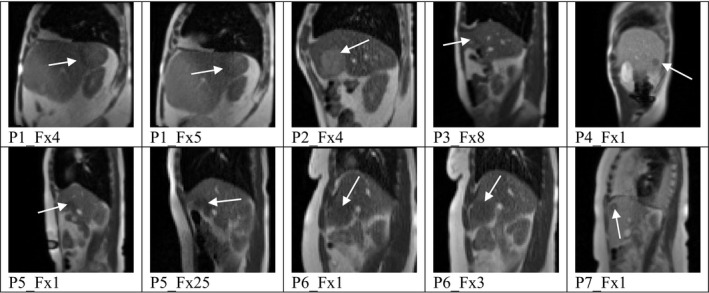
Patients and tumor locations, indicated by the white arrow, within the liver.

Two‐dimensional (2D) sagittal patient image datasets (n = 10) from the ViewRay MRIdian system were used in this study in accordance with an institutionally approved IRB protocol. This set includes seven unique patients, where two fractions from different days were used for three of the patients. All but one patient was coached to perform repeated breath‐holds during the treatment. Figure 1 displays images from each of the ten datasets used with the tumor position indicated by the white arrow. A naming convention was adopted to identify each dataset as follows: “PA_FxB”, where “A” is the arbitrary patient number and “B” is the actual fraction used. In order to meaningfully relate two fractions of the same patient, it was determined to be important to maintain the fraction number. Each image dataset displayed a visible tumor and at least one visible vessel for 175 s (700 images). The resolution of the MR image pixels was approximately 3.5 × 3.5 mm^2^. For each image dataset, the tumor and at least one vessel was tracked using a 2D tracking algorithm.^22^ For five datasets (three patients), two vessels were tracked. Manual annotations of the patient abdomen surface and diaphragm were also recorded in order to quantify motion correlation of liver tumors to the patient surface and diaphragm. The manual annotations for the patient surface could be localized to one‐half pixel as the manual annotations are based on the pixel intensity difference between the bright patient surface and dark background. Prescribing the position of the patient surface to a resolution beyond one‐half pixel would be disingenuous to what could be determined with the data. In contrast, the tracking algorithm calculates the vessel centroid position as the geometric centroid of the vessel contour in each image. We determined that rounding the centroid position to the nearest tenth of a pixel was an appropriate compromise between the surface precision of one‐half pixel and the arbitrary centroid position.

### 2.B Tumor and vessel tracking

A 2D tracking algorithm incorporating block matching and multiple simultaneous templates was utilized for efficient tracking of the tumor and vessels.^22^ Vessels that persisted through the entire dataset and were sufficiently large — on the order of a few pixels or more — were chosen for tracking. For the patients analyzed over multiple fractions, the same vessel(s) were chosen for tracking in each fraction. Since this work aimed to determine the feature motion as accurately as possible and was not meant to serve as a test of the tracking robustness, tracking algorithm parameters were modified to achieve the most accurate tracking for each data set. Manual annotations of the vessel and tumor centroids were performed for 10% of the frames to a resolution of one‐half pixel using ITK‐Snap and compared to the tracked results to validate that the tracked motion provided a proper estimate of the true feature motion. Additionally, a qualitative evaluation of the tracking performance was done by visually monitoring the tracking motion of each feature and ensuring there were not erroneous tracking results. The range of motion of the tumor was calculated for each dataset from the tracking results by finding the difference between the maximum and minimum pixel position of the tumor centroid.

### 2.C Abdomen surface and diaphragm annotations

The surface of the abdomen exhibits a stark contrast with the air in MR images; however, the tracking algorithm is written to cater to convex closed contours as opposed to a line or point. Therefore, the abdomen surface motion was quantified manually through the entire dataset. Only the anterior–posterior (AP) motion was considered as the superior–inferior (SI) position was held constant. AP motion was quantified at the SI position of maximum AP motion, similar to the study performed by Beddar et al. which looked at the correlation between internal fiducial and external marker motion in the liver using the real‐time position management (RPM) system.^5^ The resolution for the manual annotations of the abdomen surface was one‐half pixel. The pixel position marking the end of the patient and the beginning of the air was determined quantitatively by pixel intensity value.

Similar to the abdomen surface, the diaphragm highly contrasts the lung in MR images. As the diaphragm does not provide a closed contour either, manual annotations were required. The peak point in the diaphragm was chosen to track manually in both AP and SI directions to pixel level resolution.

### 2.D Assessment of motion correlation

Two calculations were performed to assess motion correlation between the tumor and feature of interest. To address phase correlation, the Pearson product moment correlation coefficient (CC) was utilized, following the correlation method used by similar studies.^15^, ^18^, ^21^, ^31^ The Pearson CC, *r*, is defined as the covariance of two sample sets, *x* and *y*, divided by the product of their standard deviations. Values for the Pearson coefficient inclusively span from −1 to 1. Values of 1 and −1 indicate perfect positive and negative correlation, respectively. Value of 0 indicates no correlation. Only the AP motion of the abdomen surface was analyzed; therefore, the SI motion CC refers to correlation of SI motion of the tumor and AP motion of the surface.

### 2.E Intra‐ and interfraction amplitude variabilities

To supplement the Pearson coefficient, the intra‐ and interfraction variabilities of the absolute difference between the motion amplitudes of the tumor and surrogate feature were analyzed. For each time point *n*, the absolute difference calculation was completed as follows:

(1) $$a_{n}=\left|x_{n}-y_{n}\right|$$

where *x_n_* and *y_n_* are geometric centroid of the tumor to the geometric centroid of the vessel or the tracked point on the abdomen surface or diaphragm, respectively. Similar to the analysis of Quirk et al., the intrafraction variability was defined as the standard deviation of the amplitude differences for all time points across the image set.^32^ This metric was evaluated to understand the degree to which the feature motion resembled the tumor motion. Comparatively, a smaller standard deviation represents smaller intrafraction variability and would suggest that the feature motion amplitude could be more accurately and directly transformed to the tumor motion amplitude. Calculations were made for each tumor and feature pair for the SI motion and AP motion individually. Note again that only the AP motion of the surface was recorded; however, AP surface motion inversely correlates to SI breathing motion. Therefore, the amplitude difference variability of the SI surface could be calculated by inverting the AP surface motion trace and comparing it to the SI tumor motion trace.

For interfraction analysis, the mean of the absolute amplitude difference results for the three patients with two fractions each was extracted from the total dataset. The error between the mean amplitude differences for each fraction was calculated:

(2) $$e=\left|\bar{a_{1}}-\bar{a_{2}}\right|$$

where $\bar{a_{1}}$ and $\bar{a_{2}}$ are the earlier and later mean amplitude differences of the two fractions from a single patient. This calculation was completed for the SI and AP results for the vessel, for the abdomen surface, and for the diaphragm. A perfectly reproduced mean amplitude difference would have error, *e*, equal to 0 mm.

Additionally, for each dataset, the breath‐hold portions of the motion traces were extracted and separately analyzed for reproducibility using these same metrics. Breath‐hold gated radiotherapy treatment involves turning the beam on during the breath‐hold phase when it is assumed the tumor position remains relatively steady. By focusing on breath‐hold data, the reproducibility of target position within the radiation field can be assessed. P4_Fx1 was not treated under breath‐hold conditions; therefore, this patient was excluded from the breath‐hold only intra‐ and interfraction amplitude variability analyses.

## 3 RESULTS

**Fig. 2:**
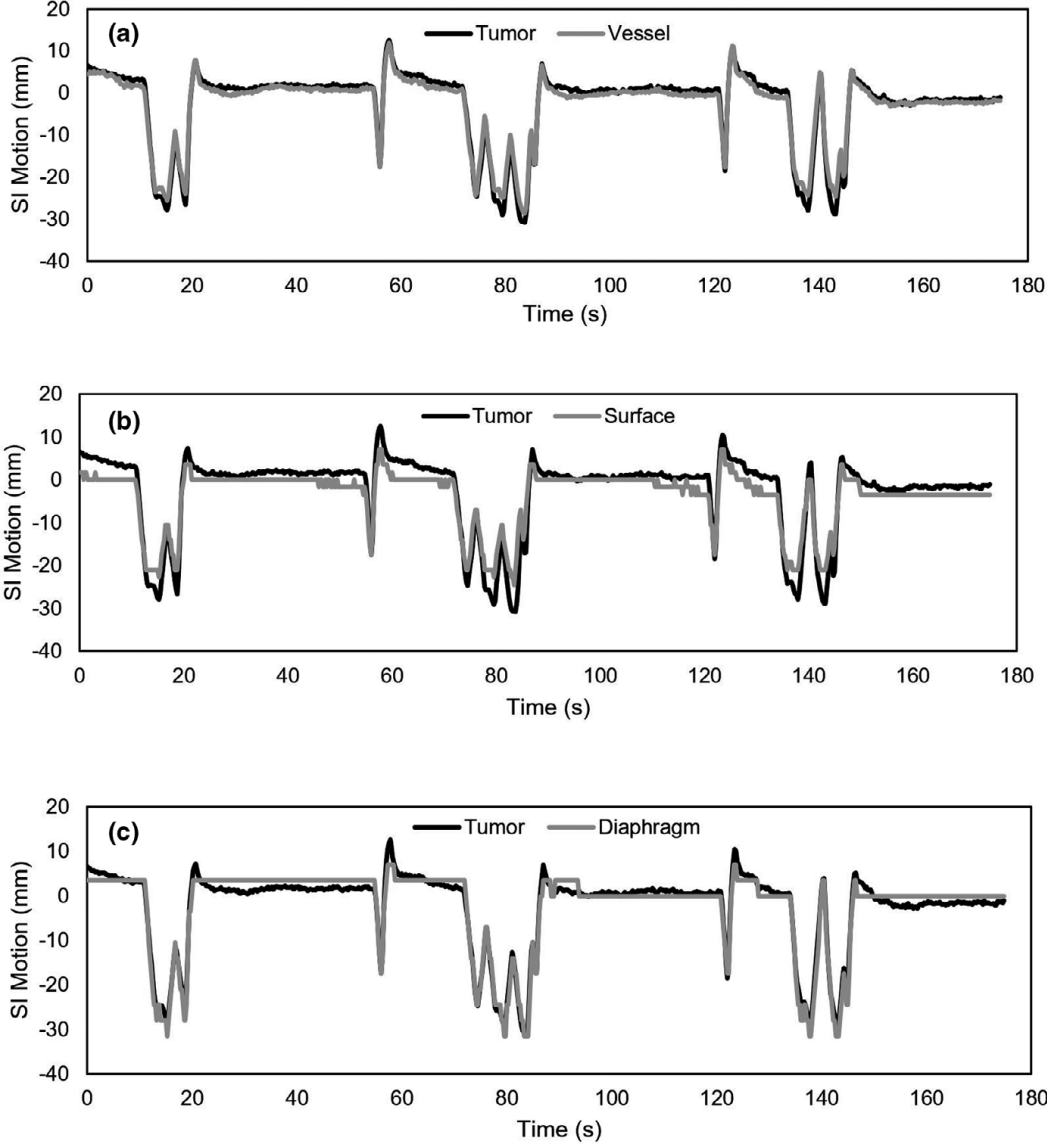
SI motion traces (mm) as a function of time for P5_Fx1 comparing the tumor to the vessel (a), to the patient surface (b), and to the diaphragm (c).

We achieved mean tracking errors relative to manual annotations between 0.28 and 1.08 pixels (0.99–3.79 mm). Figure 2 shows the motion traces for each feature as compared to that of the tumor for a representative dataset. For all datasets, all vessels analyzed were closer to the tumor than the abdomen surface. The average tumor range of motion including all patients was 22.6 ± 11.2 mm and 34.2 ± 14.1 mm in the AP and SI directions, respectively. For six out of seven patients, the SI range of motion was larger than or approximately equal to (within 1 mm) the AP range of motion. The tumor of P4_Fx1 had a notably small range of motion: 2.4 mm (AP) and 8.8 mm (SI).

### 3.A Pearson correlation coefficients

**Fig. 3:**
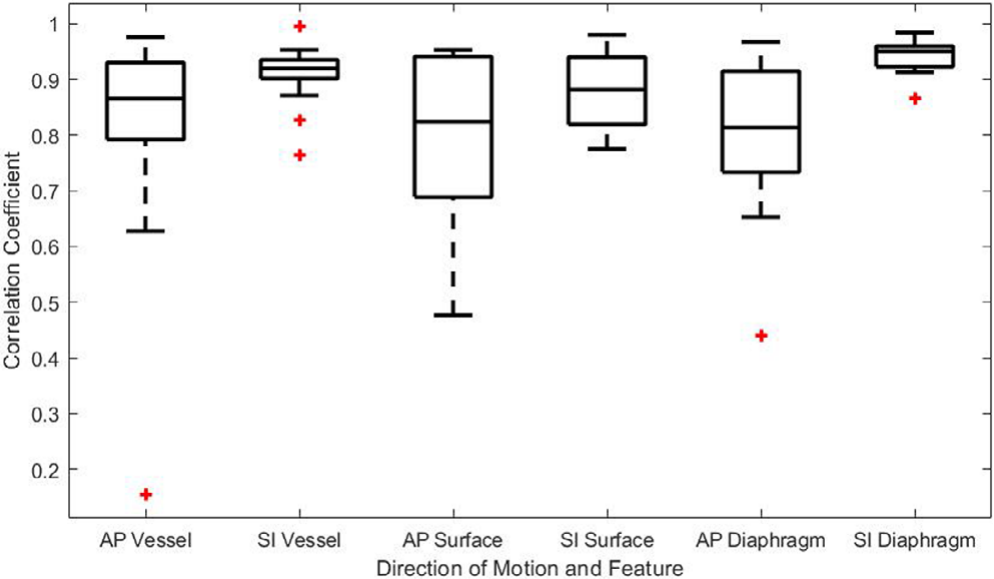
Absolute Pearson correlation coefficient results for the motion of all features in both anterior–posterior and superior–inferior directions for all datasets.

The Pearson CCs for all datasets are shown in Fig. 3. The mean CC for the vessels was 0.81 ± 0.20 and 0.91 ± 0.05 for AP and SI motion, respectively. The mean CC for the abdomen surface was 0.80 ± 0.15 and −0.88 ± 0.07 for AP and SI motion, respectively. The mean CC for the diaphragm was 0.80 ± 0.17 and 0.94 ± 0.03 for AP and SI motion, respectively. Statistical analysis showed these differences are not significant (*P* > 0.05). P4_Fx1 displayed irregular motion correlations and was considered an outlier due to the fact that the CC were very low for both the vessel and surface. For the vessel, the CCs were 0.16 and 0.76 for AP and SI motion, respectively. For the surface, the CCs were 0.48 and −0.78 for AP and SI motion, respectively. For the diaphragm, an AP motion CC could not be calculated as the diaphragm did not appear to move in the AP direction, however, the SI motion CC was calculated to be 0.92. Excluding the results from P4_Fx1, the mean CC of the dataset for the vessels increase to 0.85 ± 0.11 (AP) and 0.92 ± 0.04 (SI). The mean CC for the surface increase to 0.83 ± 0.11 (AP) and −0.89 ± 0.06 (SI). A mean CC difference of 0.10 ± 0.10 was noted between fractions from the same patient averaged across all features in both directions of motion. The mean CC difference for each feature was comparable.

### 3.B Intra‐ and interfraction amplitude variabilities

**Fig. 4:**
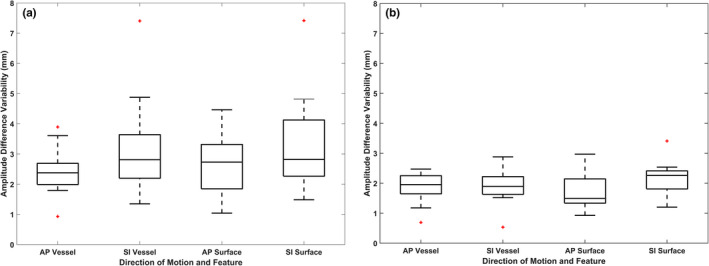
The intrafraction amplitude variability given for the anterior–posterior and superior–inferior directions for tumor to vessel and for tumor to abdomen surface for the entire length of the datasets (a) and for only the breath‐hold portions of the series (b).

Intrafraction amplitude variability results for all datasets are summarized in Table 1 as the mean and standard deviation (SD) for each feature and each direction of motion. Intrafraction amplitude variability for the vessel and patient surface is shown in Fig. 4(a) in the form of the standard deviation of the amplitude difference for each feature and direction of motion relative to the tumor. Generally, the feature to tumor intrafraction amplitude difference variability was comparable for each patient. The intrafraction amplitude variability for the breath‐hold portions of the datasets is shown in Fig. 4(b). Overall, the intrafraction variability decreased for all three features when only the breath‐hold portions were analyzed.

**Table 1:** Intrafraction amplitude variability for all datasets at all‐time points, repeated for breath‐hold only portions.

	AP vessel	SI vessel	AP surface		SI surface	AP diaphragm	SI diaphragm
Intrafraction variability (mm)							
Mean	2.47	3.14		2.70	3.43	2.76	2.91
SD	0.77	1.49		1.08	1.73	1.41	1.38
Intrafraction variability: breath‐hold (mm)							
Mean	1.88	1.92		1.72	2.19	2.13	1.87
SD	0.50	0.55		0.66	0.62	0.58	0.41

Interfraction amplitude variability results for all datasets are summarized in Table 2 as the mean and SD for each feature and each direction of motion. In contrast to the intrafraction amplitude variability analysis, we do not see a general decrease in interfraction variability when limiting the dataset to only the breath‐hold portions. The interfraction variability for both the diaphragm and surface in the AP direction of motion showed large mean values with large standard deviations. This result was also observed for the breath‐hold analysis.

**Table 2:** Interfraction amplitude variability for all datasets at all‐time points, repeated for breath‐hold only portions.

	AP vessel	SI vessel	AP surface	SI surface	AP diaphragm		SI diaphragm
Interfraction variability (mm)							
Mean	3.08	2.51	10.03	3.12		6.24	1.55
SD	1.93	1.25	6.29	2.28		2.66	0.22
Interfraction variability: breath‐hold (mm)							
Mean	2.40	2.54	11.55	1.93		7.96	0.96
SD	2.10	1.03	7.05	1.98		3.51	0.49

## 4 DISCUSSION

Overall, the tumor motion was well correlated to the vessel, diaphragm, and abdomen surface motion. In six out of ten datasets, vessels that were tracked resulted in a higher correlation with the tumor than the abdomen surface for both AP and SI motion. In three of the remaining four datasets, vessel motion had higher correlation values than the abdomen surface for one of the two directions of motion. For one patient, the abdomen surface motion more highly correlated to the tumor motion for both AP and SI motion. The diaphragm motion correlated well to tumor motion and displayed CC values comparable to the vessel. In most cases investigated, the vessels and diaphragm provided better or comparable correlation to tumor motion than the abdomen surface, and therefore may be suitable as surrogates for tumor motion in the liver for clinical use. These vessel results agree with similar findings in a conference abstract by Schlosser et al.^21^

As mentioned earlier, the abdomen surface and diaphragm displacement of P4_Fx1 was more highly correlated than the vessel displacement to the tumor displacement. It was observed that the tumor range of motion for P4_Fx1 was limited in both the AP and SI directions of motion — 2.4 and 8.8 mm, respectively. Given that the ViewRay MRIdian images have pixel sizes of 3.5 × 3.5 mm^2^, noise and uncertainty in the motion trace have greater relative effects on analysis for P4_Fx1 than for patients with larger tumor motion. Subsequently, neither the motion of the vessel nor of the abdomen surface was well correlated to that of the tumor. Interestingly, no AP motion was observed for the diaphragm, but for SI motion a high correlation value (0.92) was calculated for this patient. Additionally, this was the only patient treated with free‐breathing, implying that real‐time motion tracking of the tumor may not have been essential for treatment. For future work, the use of image data with higher resolution would allow for more stringent tracking error goals. Motion traces with lower tracking errors would minimize erroneously recorded positions in the data and could result in better correlation results.

For all cases studied, the vessel and diaphragm were closer to the tumor than the abdomen surface. This may have contributed to the increase in motion correlation especially for deep‐seated tumors. Studies indicate that motion correlation for liver tumors may be negatively impacted by increasing distances between the features of interest.^5^, ^13^ Interestingly, for the vessel dataset as a whole, no trend or relationship was observed between the average distance between the vessel and tumor and the motion CC. It was also observed that the intrafraction amplitude variability between the tumor and feature was comparable between all features further indicating that the vessel and diaphragm mimics the tumor motion as well as the patient surface.

The Pearson product moment CC has been used in previous surrogate motion studies^15^, ^18^, ^21^, ^31^; however, the Pearson coefficient has its limitations.^33^ Specifically, this metric is sensitive to outlier data since it is dependent on a mean.^33^ The use of the tracking algorithm introduces a mean tracking error of about 1 pixel or less. In the frames with tracking errors on the order of 1 pixel (3.5 mm), the true CC may be altered. Adler and Parmyrd compared the Pearson CC with the Mander's overlap coefficient for quantifying colocalization and concluded that the Pearson method is superior.^34^ Therefore, while the Pearson CC is sensitive to outlier data, it is still considered a robust method for this type of motion correlation.

The Pearson CC encompasses information about the phase of the features relative to each other. In order to further analyze amplitude relations between the features, the amplitude variability between the tumor and the vessel, abdomen surface, or diaphragm was analyzed. In general, the intrafraction amplitude variability was relatively small and comparable between the features examined. When the breath‐hold portions of the datasets were extracted and analyzed, the intrafraction amplitude variability decreased slightly. In breath‐hold gated radiotherapy, the beam is turned on during the breath‐hold portions of the treatment when the tracked object is in a user‐defined gating window. This metric shows that during these sequences the vessel‐tumor amplitude relationship is stable. Both of these results show that the vessel is a consistent surrogate for intrafraction tumor motion. More complex amplitude relationships were not investigated as part of this study which could have resulted in an even more suitable relationship between the features and tumor.

Furthermore, the mean amplitude differences were compared for two fractions of the same patient for the interfraction investigation of the amplitude variability. Again, the breath‐hold portions of the dataset were extracted and analyzed on their own. An overall decrease in variability was not observed when only the breath‐hold portions were examined. While the variability was on the order of a few mm for the vessel in both directions and the surface and diaphragm for the SI direction, the variability was much larger for the tumor‐surface and tumor‐diaphragm relationship in the AP direction. These results illustrate the interfraction changes in the patient and the importance of set‐up repeatability when using an external surrogate marker. Additionally, creating a new model for the relationship between the tumor and surrogate feature may be necessary to reduce the uncertainty introduced between treatment fractions.

This work was limited to a 2D motion analysis only for the superior–inferior and anterior–posterior directions of motion. The left–right motion was not included in this study; therefore, it cannot be claimed that vessel and tumor motion were fully captured. However, previously performed studies observed minimum left–right motion of abdominal tumors: 1–2 mm or less,^6^ and up to 3 mm.^35^ Additionally, AAPM Task Group 76 highlights SI motion for the liver, while acknowledging limited motion in the left–right plane: 2 mm or less.^36^ Recently, Park et al used 4D MR to track lung tumors and external surrogate markers showing that external surrogates generally correlate well with tumor motion, however, there is often a systemic phase mismatch as well as cycle‐to‐cycle variation that went undetected by the external surrogate.^7^ Park et al acknowledges that a challenge of their retrospective 4D MR reconstruction is the introduction of artifacts in the lateral plane that may affect the integrity of their 3D correlation results.^7^ Considering the small lateral motion reported and the challenges of 3D MR reconstruction, it is believed that this study is valuable without an analysis of the motion in the lateral plane. Future studies could build on this work by investigating the liver tumor and vessel motion correlation in the lateral plane independently or in 3D.

The number of patients included in this study was limited by the number of liver patients treated at our institution under the IRB protocol allowing researchers to retrospectively analyze image data. Additionally, a vessel must remain visible throughout the image sequence analyzed in order to acquire a full motion trace to compare to the tumor motion. The plane of view observed during the ViewRay treatment was not chosen with this study in mind; therefore, multiple patient datasets lacked a qualifying vessel. Furthermore, the plane analyzed is not necessarily ideal for this work; there may be a better plane from which to view both the tumor and vessel for improved correlation results. Similarly, the abdomen surface point selected for tracking is in the same lateral position as the tumor. This position for the abdomen surface is ideal and may not be practical in the clinic for systems such as the Varian RPM that includes a plastic box to sit on the patient. Depending on the location of the tumor, external surrogate placement closer to the midline of the patient abdomen may increase distance between the tumor and the surrogate and decrease the motion correlation.^16^ Fayad et al^16^ showed that external motion correlation results are dependent on the external surrogate placement. Overall, the impact of target tracing accuracy, image resolution, and patient cohort size warrants further investigation.

Indirect tumor tracking in the liver may be necessary for cases where the tumor does not provide sufficient contrast with surrounding tissue or when alternative image guidance methods such as ultrasound are employed. In these cases, liver vessels may provide suitable surrogates for motion as the liver is highly vascular and vessels are differentiable from liver tissue in both MR and ultrasound. Bloemen‐Van Gurp et al confirms that the use of alternative anatomy such as vessels was “indispensable” for cases where the lesion was not visible or was too large to fit in the ultrasound field of view.^29^ With growing interest in accurate target tracking and localization, multiple groups have dedicated time and energy to develop sophisticated tracking algorithms for liver vessel tracking.^22^, ^23^, ^24^, ^25^, ^26^, ^27^ Similarly, groups have utilized the vasculature of the liver to develop motion tracking and prediction algorithms.^28^, ^30^ Preiswerk et al^28^ used features of the liver such as vessels and boundaries to create a model to predict liver motion and Akino et al^30^ used liver vessels to validate a motion vector‐based tumor motion algorithm, thus assuming vessel and tumor motion correlates in the liver. Yet, even with the development of these innovations, to the best of our knowledge there have been no studies reporting on the correlation between liver vessel and tumor motion with patient data until now. Additional work will explore the feasibility and reproducibility of imaging and tracking vessels in the liver with a simultaneous MR and ultrasound system. While the datasets available for this study did not provide the necessary information to assess the dosimetric impact of using vessels as motion surrogates, future work will assess this clinical impact.

## 5 CONCLUSIONS

The abdomen surface, liver vessel, and diaphragm motion relationship to liver tumor motion was successfully analyzed using multiple metrics to investigate the general phase and amplitudes of motion. Tumor motion in the liver was well correlated to abdomen surface, vessel, and diaphragm motion. The tumor motion can be captured with a direct relationship with the vessel motion relatively well as indicated by the results of the intrafraction amplitude variability analysis. The results of this study suggest that the diaphragm and vessels in the liver are suitable surrogates for liver tumor motion. While out of the scope of this work, an additional investigation into the dosimetric impact of utilizing liver vessels as surrogates of liver tumor motion is warranted. Finally, taking the results of this study forward, efforts to evaluate the practicality of tracking vessels in the clinic utilizing different motion management solutions such as MR and ultrasound are currently underway at our institution.

## CONFLICT OF INTEREST

No conflict of interest.
